# Genome-wide characterization and expression analysis of the *CINNAMYL ALCOHOL DEHYDROGENASE* gene family in *Triticum aestivum*

**DOI:** 10.1186/s12864-024-10648-w

**Published:** 2024-08-29

**Authors:** Luigi M. Peracchi, Rhoda A.T. Brew-Appiah, Kimberly Garland-Campbell, Eric H. Roalson, Karen A. Sanguinet

**Affiliations:** 1 Department of Crop and Soil Sciences, Washington State University, Pullman, WA 99164 USA; 2 Molecular Plant Sciences Graduate Group, Washington State University, Pullman, WA 99164 USA; 3 USDA-ARS Wheat Health, Genetics and Quality Research, Pullman, WA 99164 USA; 4 School of Biological Sciences, Washington State University, Pullman, WA 99164 USA

**Keywords:** CAD, Bread wheat, Lignin, Biotic stress, Genomics

## Abstract

**Background:**

CINNAMYL ALCOHOL DEHYDROGENASE (CAD) catalyzes the NADPH-dependent reduction of cinnamaldehydes into cinnamyl alcohols and is a key enzyme found at the final step of the monolignol pathway. Cinnamyl alcohols and their conjugates are subsequently polymerized in the secondary cell wall to form lignin. *CAD* genes are typically encoded by multi-gene families and thus traditionally organized into general classifications of functional relevance.

**Results:**

*In silico* analysis of the hexaploid *Triticum aestivum* genome revealed 47 high confidence *TaCAD* copies, of which three were determined to be the most significant isoforms (class I) considered *bone fide CADs*. Class I CADs were expressed throughout development both in RNAseq data sets as well as via qRT-PCR analysis. Of the 37 class II *TaCADs* identified, two groups were observed to be significantly co-expressed with class I *TaCADs* in developing tissue and under chitin elicitation in RNAseq data sets. These co-expressed class II *TaCADs* were also found to be phylogenetically unrelated to a separate clade of class II *TaCADs* previously reported to be an influential resistance factor to pathogenic fungal infection. Lastly, two groups were phylogenetically identified as class III *TaCADs*, which possess distinct conserved gene structures. However, the lack of data supporting their catalytic activity for cinnamaldehydes and their bereft transcriptional presence in lignifying tissues challenges their designation and function as CADs.

**Conclusions:**

Taken together, our comprehensive transcriptomic analyses suggest that *TaCAD* genes contribute to overlapping but nonredundant functions during *T. aestivum* growth and development across a wide variety of agroecosystems and provide tolerance to various stressors.

**Supplementary Information:**

The online version contains supplementary material available at 10.1186/s12864-024-10648-w.

## Background

Hexaploid (AABBDD, 2n = 6x = 42) *Triticum aestivum*, or bread wheat, is a critical cereal for global food security due to its popularity as a human food grain and ability to tolerate a large range of temperatures, soil conditions, and photoperiods [1, 2]. However, increasingly unpredictable weather patterns are beginning to threaten projected *T. aestivum* productivity to maintain current steady-state food production [3, 4]. Bread wheat grown via dryland farming is particularly vulnerable to the effects of changing weather patterns, where the lack of irrigation infrastructure and monocrop cultivation over vast areas make detection and management of disease difficult [5–9]. Thus, there is a pressing need to research novel stress resistance traits that can be integrated into existing premium wheat lines.

Lignin is progressively garnering attention for its significant contributions to both abiotic stress tolerance [10–14] and pathogen resistance [15–22]. Grass lignin is a heterogeneous phenolic polymer primarily composed of *p*-coumaryl alcohol (H), coniferyl alcohol (G), sinapyl alcohol (S), *p*-coumaric acid, ferulic acid, and the flavone tricin [23–26]. Numerous studies perturbing the monolignol synthesis pathway have led to the current theory that monolignol synthesis is a partial but significant point of control that governs lignin polymer compositions [27]. This theory is supported by functional analysis of the last phenylpropanoid enzyme, CINNAMYL ALCOHOL DEHYDROGENASE (CAD) in grasses including *Brachypodium distachyon* [28–30], *Oryza sativa* [31–34], *Panicum virgatum* [35], *Sorghum bicolor* [36–40], and *Zea mays* [41–43]. CAD is a Zn^2+^ binding NADPH-dependent homodimer responsible for converting cinnamaldehydes to H, G, and S lignin monomers [44, 45].

Although *CAD* perturbation significantly reduces H, G, and S monomer synthesis resulting in a significant increase of cinnamaldehyde integration into the lignin polymer [43], H monomers were recently found polymerized in *Arabidopsis thaliana Atcadc/d* null mutant lignin, suggesting a H monolignol synthesis pathway independent of CAD activity [46]. *CAD* perturbation phenotypes include reduced plant height, delayed flowering, and a conspicuous brown midrib in C4 grasses such as *S. bicolor* [36, 37, 47], *Z. mays* [41, 43, 48, 49], and *P. virgatum* [38]. Interestingly, *CAD* perturbations in C3 grass *B. distachyon* do not display a brown midrib phenotype but were identified based on a red culm phenotype [29]. Increased polymerization of cinnamaldehydes into the lignin polymer is currently hypothesized to increase cell wall carbohydrate exposure to chemical and biological degrading agents [43, 50, 51], but the mechanisms underlying the mutant growth phenotypes are poorly understood.

Phylogenetic analyses have been used to organize CAD families into three general classes [28, 52–54]. Class I CADs are typically the dominant active isoform found in and around lignifying tissues, possess a highly conserved primary sequence structure across most species, and have been extensively studied as a target to improve saccharification of biomass for biofuel production [29, 34, 38, 39, 42, 43, 55–60]. Class II CADs are a much larger and more diverse group of CAD isoforms. Some class II CADs have been found to be incidentally co-expressed with class I CADs and can reduce cinnamaldehydes [39, 54, 61]. Class II CAD sequence diversity may imply some class II CADs contribute uncharacterized sub-functionalized roles beyond cinnamaldehyde reduction, as class II CADs have been observed to modulate transcription of defense-related genes as well as other genes in the phenylpropanoid biosynthetic pathway [62, 63]. Lastly, class III CADs possess highly conserved sequence structure, but have been observed to have little to no cinnamaldehyde catalytic activity [54, 64, 65]. Despite having high sequence conservation and near ubiquitous but low amounts of transcript detected in most tissue types, class III CAD function is still largely uncharacterized.

The *TaCAD* gene family has been previously investigated, revealing 11 putative *TaCAD* gene copies, one of which was identified as *TaCAD1* [66]. Another study identified Class II *TaCAD12* as being differentially upregulated in a sharp eyespot-resistant *T. aestivum* line after infection. Furthermore, overexpression analysis of *TaCAD12* resulted in a concomitant upregulation of defense related genes (*Defensin*,* PR10*,* PR17c*, and *Chitinase1*) and phenylpropanoid genes (*TaCAD1*,* TaCCR*, and *TaCOMT1*). Recently, the full coding sequence of *TaCAD1* was cloned and determined to significantly contribute to lodging resistance [67]. The size and complexity of the *T. aestivum* genome has limited past attempts to thoroughly investigate the *TaCAD* gene family, thus limiting efforts to identify and functionally characterize *TaCAD* genes that may significantly influence development and stress resistance. However, the publicly available high resolution hexaploid *T. aestivum sp. Chinese spring* (CS) genome assembly [68] has enabled a thorough reexamination of the *TaCAD* gene family. The present study identifies 47 unique *TaCAD* genes, which were subsequently characterized through rooted protein phylogeny, gene structure analysis, and protein sequence alignment with previously characterized and *bona fide* CADs. The data presented in this study provides a comprehensive identification and partial characterization of the *TaCAD* gene family, which may contribute to downstream identification of novel lignin-based stress responses that support and enhance wheat cultivation in vulnerable agroecosystems.

## Results

### Identification and classification of the *T. aestivum CAD gene family*

**Fig. 1:**
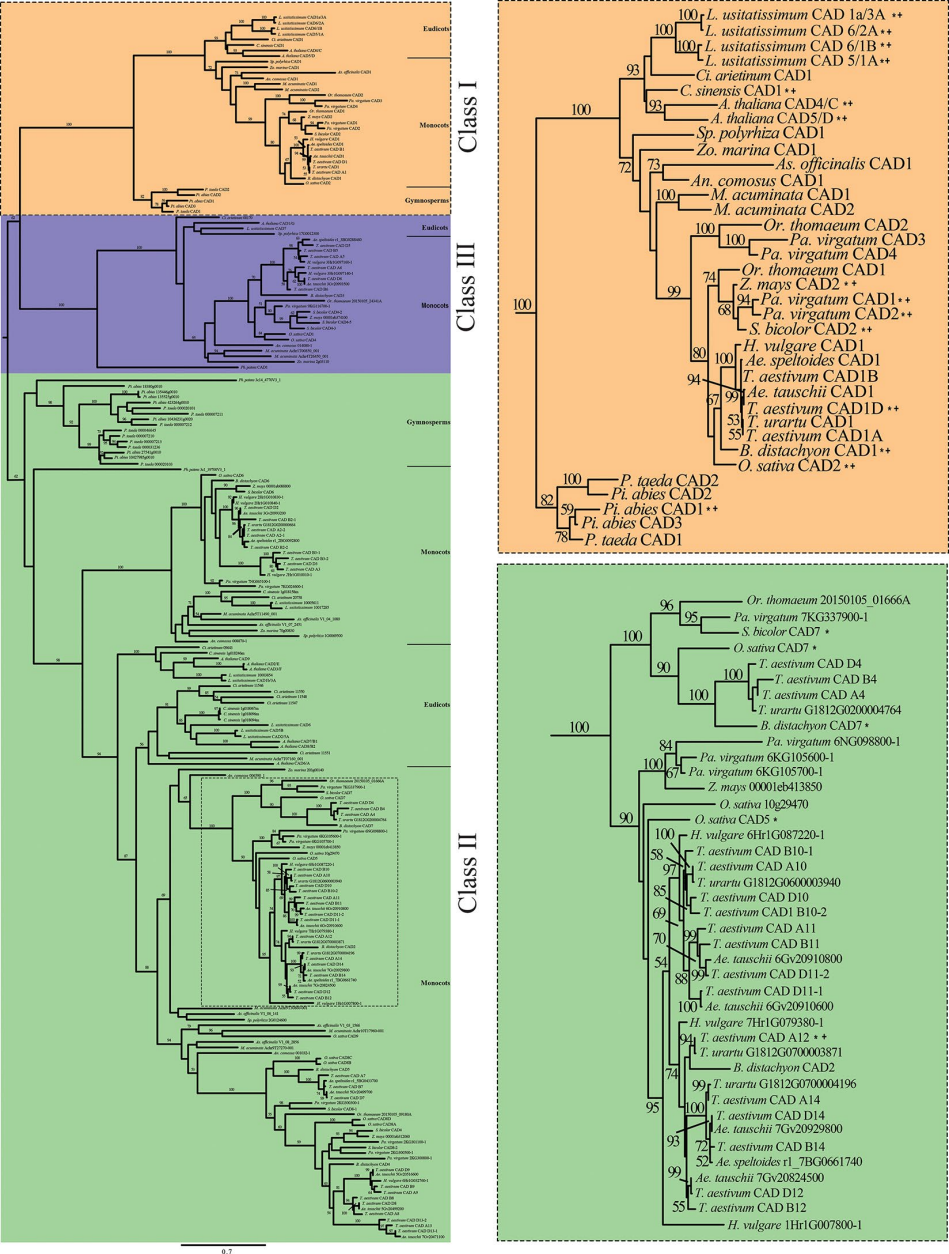
Phylogenetic relationships of CAD protein sequences derived from gymnosperm, monocot, and dicot species. Orange box represents class I, green box represents class II, and blue box represents class III. Numbers depict confidence intervals for the phylogenetic association. Class I, II, and III clades appear to organize into gymnosperm, eudicot, and monocot subclades. CADs that have been previously identified are annotated with a “*”, whereas CADs that have been previously functionally characterized are annotated with a “+”

To provide insight into the relationships among the major classes of *CAD* genes and copies of those genes within classes, we performed maximum likelihood (ML) phylogenetic analysis of the 219 amino acid sequences derived from a diverse selection of gymnosperms, eudicots, and monocots (Table S1), which resulted in one best tree (logL: -50162.405459) with strong support for branching structure as measured by bootstrap values (Fig. 1). The three recognized classes are monophyletic when rooted between the class II clade and the class I + class III clade, and these classes are suggested to have originated at a minimum prior to the diversification of seed plants, but likely (at least for the earliest duplication) near the base of land plants prior to the divergence of bryophytes and vascular plants. Since no green algal species were included in this analysis we cannot assess if any duplications predated the move of plants to land. Multiple duplications of the gene family have also occurred within each of the classes and there is significant variability in the number of gene copies in different species, at least of those included in this analysis.

**Fig. 2:**
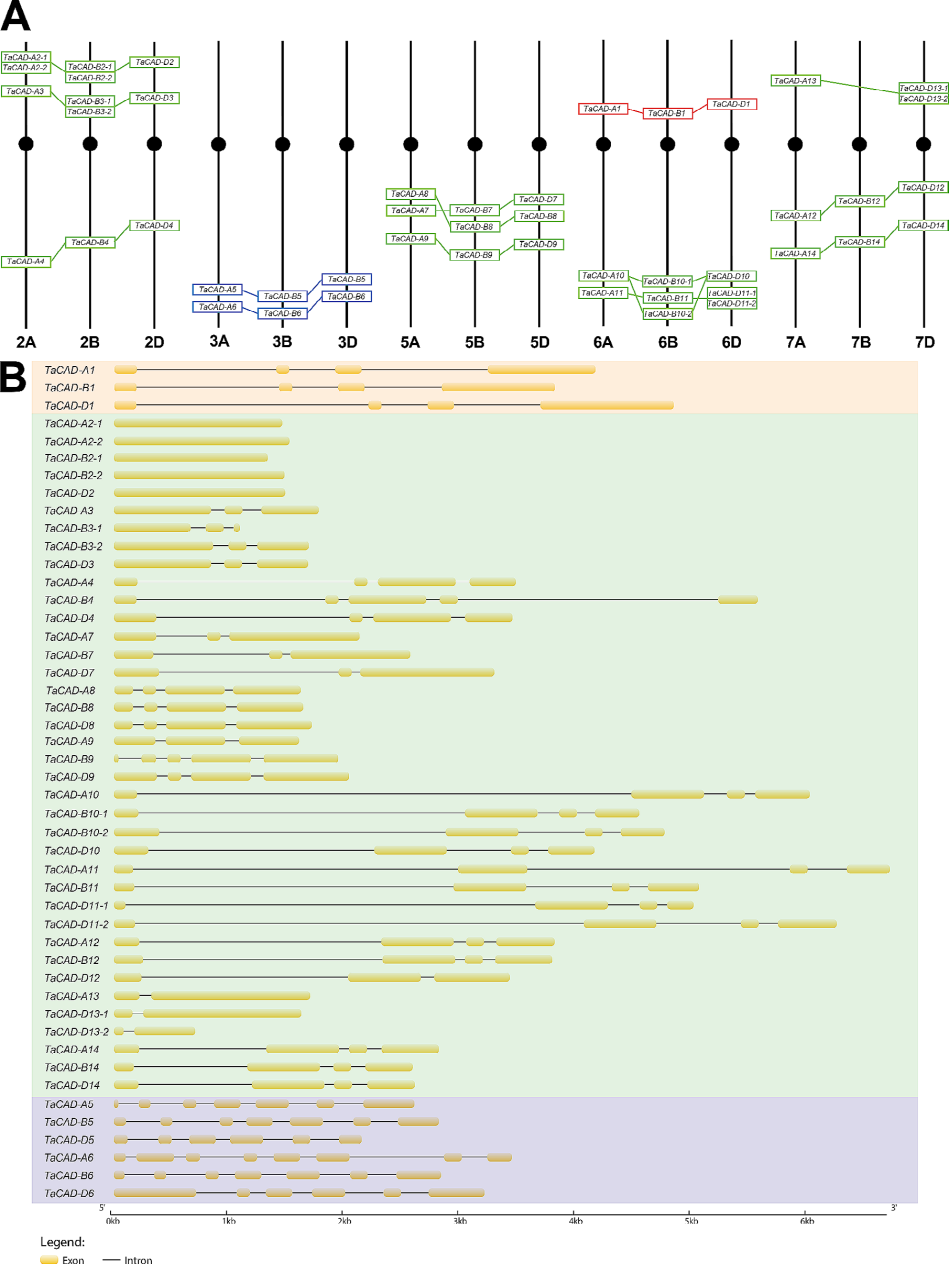
*TaCAD* homoeolog distribution and gene body morphologies. (**A**) Distribution of *TaCAD* homoeologs on the hexaploid *T. aestivum* chromosomes. Orange boxes represent class I, green boxes represent class II, and blue boxes represent class III. Lines connecting boxes represent phylogenetically determined homoeologous relationships. Boxes touching represent predicted duplication events. Depicted gene locations are estimated. (**B**) *TaCAD* gene body morphologies. Exons are depicted as yellow boxes, while introns are depicted as lines. Gene body sizes are to scale, as denoted by the ladder depicting number of base pairs given distance. *TaCAD* gene bodies are organized by phylogenetic association, and homoeologous relationships

The rooted phylogeny is organized into three distinct phylogenetic clades closely resembling previous CAD genome-wide phylogenetic analyses [52, 53, 61, 69]. All previously *bona fide* class I CAD protein sequences were found in a single clade, along with three class I TaCADs, one of which was previously characterized and named TaCAD1 [66, 67], here honorifically designated TaCAD-D1 due to its annotation on the short arm of the 6D chromosome (Fig. 2a). In support of TaCAD-D1 annotation on the 6D chromosome, TaCAD-D1 was found to phylogenetically neighbor the D subgenome progenitor *Ae. tauschii* putative class I CAD ortholog [70]. In addition, the A and B subgenomes each appear to contribute a novel class I *CAD* homoeolog. The putative A subgenome homoeolog, here designated TaCAD-A1 is found annotated on the short arm of the 6 A chromosome, and also phylogenetically neighbors the A subgenome progenitor *T. urartu* putative class I *CAD* ortholog [71]. Similarly, the putative B subgenome homoeolog, here designated *TaCAD-B1*, is annotated on the short arm of the 6B chromosome and phylogenetically neighbors the proposed B subgenome progenitor *Ae. speltoides* putative class I *CAD* ortholog [72].

An EST database search and subsequent phylogenetic alignment concluded TaCAD2 (TC143210) and TaCAD4 (TC144004) were closely related to TaCAD1(TaCAD-D1) [66], suggesting that TaCAD2 and TaCAD4 may be either TaCAD-A1 or TaCAD-B1. Previous *in silico* protein sequence analysis of TaCAD2 reported 40 missing amino acids on the C-terminus when aligned with TaCAD1(TaCAD-D1) [66]. However, *in silico* sequence analysis predicts all three class I TaCAD homoeolog protein sequences are 360 amino acids in length (Table S2). TaCAD4 protein sequence was previously reported to be 219 amino acids in length [66], which does not match any sequence length of any other TaCAD isoform presented in this study. Thus, the relationship between TaCAD2 and TaCAD4 to either TaCAD-A1 or TaCAD-B1 cannot be resolved as the sequences from the previous analysis are no longer available and not reported in the literature (Ma et al., 2010).

*TaCAD12* was previously identified and characterized [62]; therefore, *TaCAD12* is also given an honorific designation of *TaCAD-A12*, phylogenetically organized into the class II subclade (Fig. 1), and is found annotated on the long arm of chromosome 7 A (Fig. 2A). *TaCAD-A12* is phylogenetically grouped with two homoeologs: *TaCAD-B12* on the long arm of chromosome 7B; and *TaCAD-D12* on the long arm of chromosome 7D.

The remaining TaCAD homoeolog groups were given designations based on subgenome annotation, appearance relative to the 5’ telomeric region, chromosome number, and phylogenetically determined duplications (Fig. 2A). Several *TaCAD* copies annotated near 5’ and 3’ telomeric regions appear to be products of recent duplication events based on the phylogenetic hypothesis. Conflict with gene order and homoeologous associations seen on chromosomes 5 and 6 are indicative of recombination events. For instance, annotated *TaCAD* gene order among class II *TaCAD-A7* and *TaCAD-A8* on chromosome 5 A conflict with phylogenetic associations (Fig. 1), suggesting a recombination event likely occurred here. A similar conflict with gene location and phylogenetic relationship is found on chromosome 6B, where a recombination event likely transposed the genomic locations *TaCAD-B10-2* and *TaCAD-B11*. Class II *TaCAD-13* copies are annotated on the short arm of chromosomes 7 A and 7D, yet a *TaCAD-13* homoeolog is not annotated on short arm of chromosome 7B, suggesting either a deletion event may have occurred on the short arm of chromosome 7B, or a significant mutation in a hypothetical *TaCAD-B13* resulted in it failing to be identified by our search parameters. Lastly, all class III *CADs* appear to be organized on the long arms of chromosomes 3 A, 3B, and 3D, where the phylogeny suggests two distinct homoeologous triads *TaCAD-5* and *TaCAD-6*.

Gene structure analysis of the *T*a*CAD* genomic bodies reveal similarities to past characterized *CAD* families (Fig. 2B). Class I *TaCAD* genomic sequences contain four exons, each resulting in a conserved 1083 bp length coding sequence, and possess a uniquely conserved 114 bp second exon that codes for a substrate binding motif. Class II *TaCAD* genomic structures have the most varied gene structure morphology of the three classes. Class II *TaCAD* exon numbers range from one to five, separated by widely varying intron lengths (Table S3). Lastly, Class III *TaCADs* have distinct gene structure morphology relative to all other *TaCADs* containing between six and eight exons: the greatest number of exons of all *TaCADs*. Lastly, not all species used to root the phylogeny contributed a class III copy.

### Major motifs and functional residues in critical domains

**Fig. 3:**
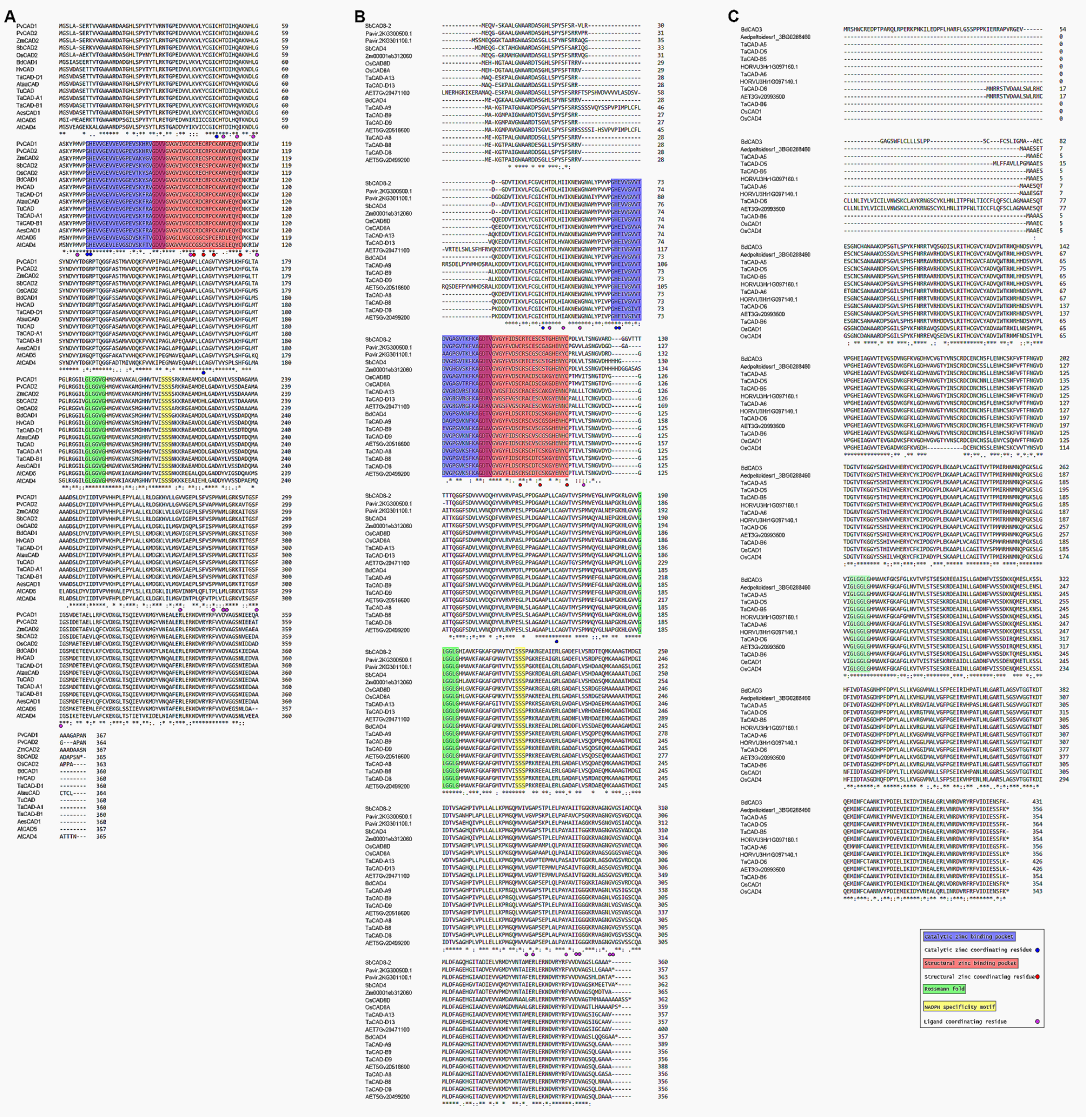
Multiple protein sequence alignments of the class I predicted catalytically active class II, and class III TaCADs (**A**) In class I CADs, two major structural motifs variants are highly conserved across all species examined: a Rossman fold GLGGVG variant, and a SSSX_2_K NADPH-specificity motif. Zinc coordinating residues and significant hydrophobic residues found in the substrate binding pocket are also highly conserved. (**B**) Class II TaCAD-8, TaCAD-9, and TaCAD-13 homoeologs phylogenetically related to *bona fide* catalytically active class II SbCAD4 possess conserved GGLGLG Rossman fold variant and SSSX_2_K NADPH-specificity motif. (**C**) Class III TaCAD amino acid sequences differ from both class I and class II TaCADs, containing a conserved GGLGLG Rossman fold variant and STSX_2_K putative NADPH-specificity motif

To further characterize phylogenetic classifications, class I, select class II, and class III TaCADs were subjected to a multiple protein sequence alignment to identify major functional motifs of previously described in *bona fide* CAD orthologs [29, 37, 39, 54, 66] (Fig. 3A). The class I TaCAD homoeologs TaCAD-A1, TaCAD-B1, and TaCAD-D1 were found to contain conserved functional motifs characteristic of the medium and short chain dehydrogenase/reductase superfamily, including a catalytic Zn^2+^ binding domain ^69^GHE(X)_2_G(X)_5_G(X)_2_V^92^, a structural Zn^2+^ domain ^89^GD(X)_9_C(X)_2_C(X)_2_C(X)_8_C^114^, and a NADP(H) binding domain GLGG(V/L)G known as a Rossmann Fold (Fig. 3A). In addition, all three class I TaCAD homoeologs contained identical locations of the catalytic Zn^2+^ coordinating residues at Cys48, His70 and Cys164, whereas structural Zn^2+^ coordinating residues are estimated to be at Cys101, Cys104, Cys107, and Cys115. The Rossmann Fold NADP(H) binding motif was found at ^189^GLGGVG^194^, along with the ^212^SSS(X)_2_K^217^ motif conserved in all aligned class I CADs, which is postulated to coordinate NADP(H) into the catalytic binding pocket [39, 45]. The class I TaCAD substrate binding pocket is reportedly composed of twelve conserved hydrophobic residues, nine of which are predicted to be located on one subunit at Thr50, Gln54, Leu59, Met66, Cys100, Trp120, Val277, Phe300, and Ile301, and three of which are predicted to be found on the opposite subunit at Pro287, Met290, and Leu29 [39]. Lastly, all monocot class I CADs appear to have both a highly conserved sequence homology, with notable amino acid substitution patterns conserved among monocot species possessing either C3 or C4 photosynthesis.

The phylogenetically predicted class II TaCADs differ from the class I CADs in several aspects, the first being protein sequence diversity. The protein sequences and subsequent molecular weights, isoelectric points and overall charge vary among predicted class II TaCADs, compared to relatively conserved values found in class I CADs (Table S2). Class II CAD functional motifs differ from class I CADs, notably in the Rossmann Fold motif, which is found to be exclusively GLGGLG, and in the variants of a S(S/T)S(X)_2_K NADP(H) specificity domain. Recent evidence suggests that some transcriptionally active *S. bicolor* class II *SbCADs* are also catalytically active [39]. To investigate if catalytically significant functional motifs and residues found in previously characterized SbCAD4 are conserved in related TaCADs, the phylogenetic clade containing SbCAD4 was subjected to a multiple protein sequence alignment (Fig. 3B). The TaCAD-8, TaCAD-9 homoeolog triad, and TaCAD-13 homoeolog pair appear to align closely with SbCAD4 and contain the SSS(X)_2_K variant NADP(H) specificity motif. Moreover, the SbCAD4 structural zinc binding domain, catalytic zinc binding domain, and the hydrophobic residues that comprise the substrate binding pocket appear to be highly conserved in the aligned TaCAD orthologs.

Class II *TaCAD-A12* was also found to be upregulated in response to sharp eyespot disease [62]. However, the exact function TaCAD-A12 contributed to the disease resistance was not clearly determined. Thus, a similar multiple protein sequence alignment was performed to determine if the class II phylogenetic clade containing TaCAD-A12 has conserved protein motifs and residues that reflect motifs and residues found in enzymatically active CAD isoforms (Figure S1). TaCAD-A12 and its homoeologs were found to possess an SSS(X)_2_K NADPH specificity motif and a GLGGLG Rossman Fold motif variant, similar to TaCAD-8, TaCAD-9, and TaCAD-13 homoeologs. Of particular note, both OsCAD5 and OsCAD7 [32] were found phylogenetically associated with the TaCAD-12 homoeologs and possess similar primary sequences.

Class III TaCADs were also aligned and analyzed (Fig. 3C). Globally, TaCAD-5 and TaCAD-6 appear to have a highly conserved sequence homology with other class III CADs, with notable amino acid substitutions in either C3 or C4 grasses. Class III *TaCADs* also possess the GLGGLG Rossmann Fold variant, but unlike class I or class II CADs, the STS(X)_2_K variant NADP(H) specificity motif was found conserved in all putative class III CADs.

### Genome-wide analysis of *TaCAD* transcript profiles during development

**Fig. 4:**
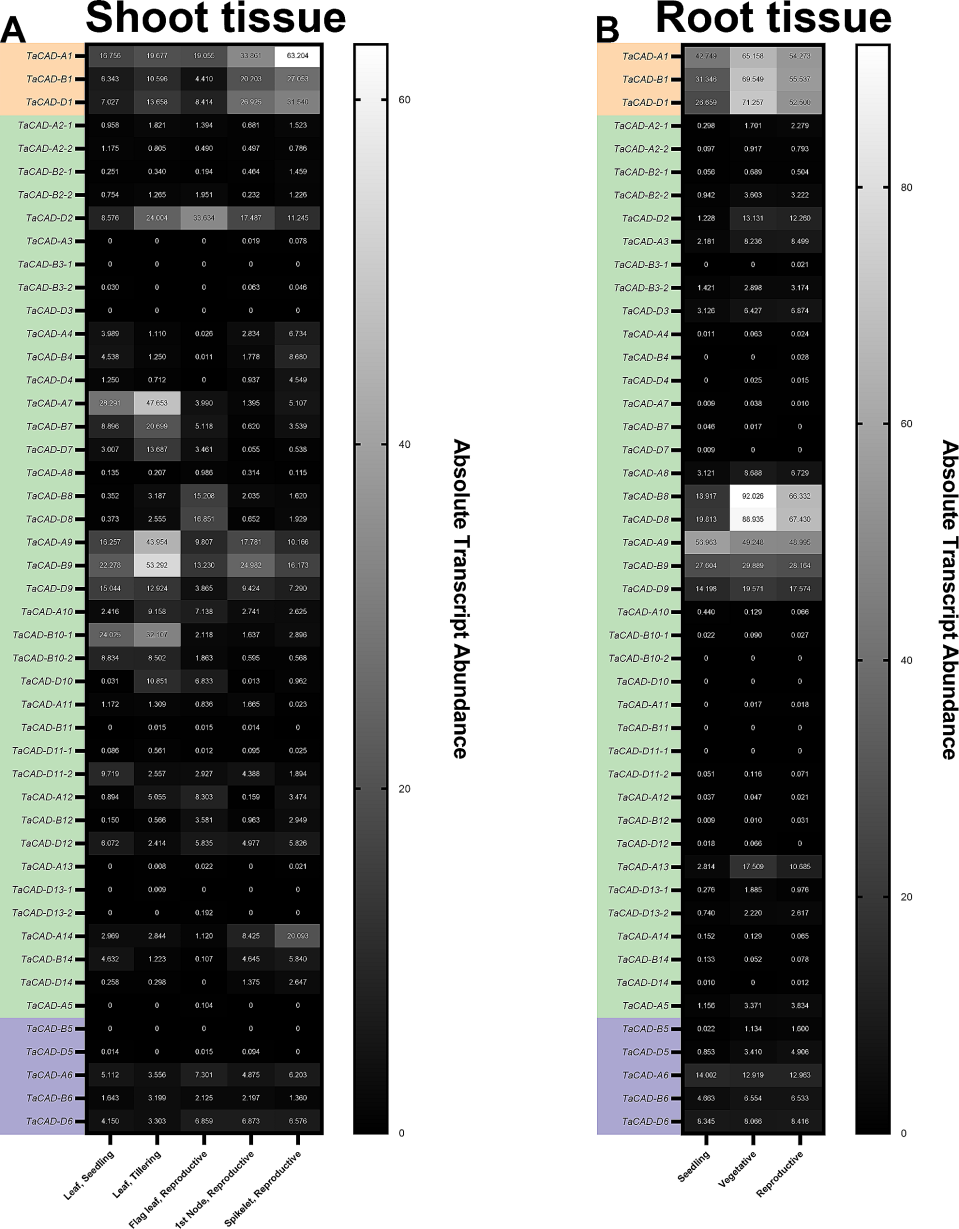
Heatmap of *TaCAD* expression, development. *TaCADs* are organized by classification and homoeologous relationships. Warmer colors denote greater transcript abundance. (**A**) Transcript abundance of *TaCADs* at seedling, tilling, and reproductive growth stages in aerial tissues. (**B**) Transcript abundance of *TaCADs* in root tissue at seedling, vegetative, and reproductive stages

In order to investigate the spatiotemporal expression patterns of the *TaCAD* gene family, the expVIP wheat transcriptome expression browser was queried for total detectable transcript at key development stages in root, leaf, node, and spikelet tissues in the Chinese Spring (CS) genetic background [73]. Class I Ta*CAD* transcript was detectable in all aerial tissues during seedling, vegetative, and reproductive development phases, and a positive correlation is seen with transcript abundance and general plant age (Fig. 4A). In addition, the class I *TaCADs* were subjected to a hierarchical clustering analysis and were found to co-express with other putative functional orthologs related to lignin biosynthesis (Figure S2). Lastly, class I *TaCAD* transcript in roots appears to be relatively steady during all development phases (Fig. 4B), but are not significantly co-expressed with other lignin synthesis genes in root tissue (Figure S3).

Class II *TaCAD-8* and *TaCAD-9* homoeolog groups were found to have comparable transcript abundance to the class I *TaCADs* at one or more development stages in roots, leaves, stems, and spikelet tissue (Fig. 4). To test if these class II *TaCADs* are co-expressed with class I *TaCADs*, the class I *TaCADs* and the *TaCAD-8* and *TaCAD-9* homoeolog groups were subjected to a hierarchical clustering analysis (Figure S2). *TaCAD-A9* is predicted to be significantly co-expressed in root tissue with *TaCAD-A1*. Regarding previously characterized class II *TaCAD-A12* and its putative homoeologs, low levels of transcript were detected. Lastly, class III *TaCAD-B5* was not detectable in aerial tissue but was detected in very low amounts at all stages of root growth, whereas the other five class III *TaCAD* homoeologs were detectable in low quantities in both aerial and root tissues.

**Fig. 5:**
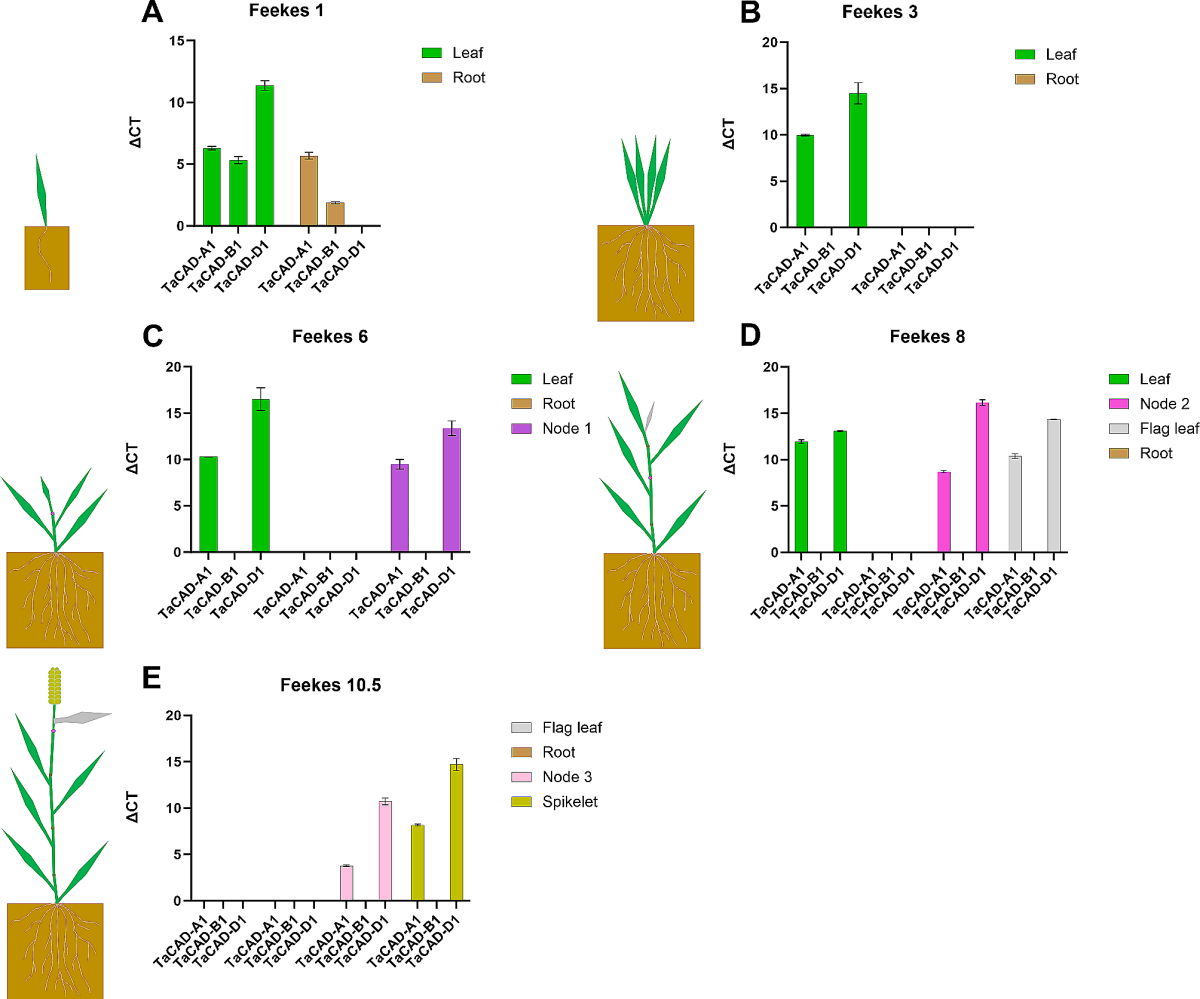
qRT-PCR of the class I *TaCADs* in different CS tissue types at different development stages. (**A**) seedling stages of development. All *TaCADs* are detectable except for *TaCAD-D1* in roots at Feekes 1 in roots. (**B**) Class I *TaCAD* transcript appears to become undetectable in roots, and *TaCAD-B1* becomes undetectable in leaf tissue (**C**) *TaCAD-A1* and *TaCAD-D1* are consistently co-expressed, while *TaCAD-B1* remains undetectable. (**D**) *TaCAD-A1* and *TaCAD-B1* transcript is detectable in developing Flag leaf at levels consistent with node and leaf tissue. (**E**) Mature flag leaf has no detectable class I *TaCAD* transcript

To validate this RNAseq dataset, homoeolog-specific primers for class I *TaCADs* were designed to test for expression in tissue and growth stages mirroring the expVIP RNAseq dataset (Table S4). Class I *TaCADs* were found detectable in both leaf and root seedling tissue but became undetectable in root tissue during vegetative and reproductive growth stages (Fig. 5). Furthermore, *TaCAD-B1* was not detectable in leaf, node, or spikelet tissue, during vegetative and reproductive development stages, whereas *TaCAD-A1* and *TaCAD-D1* were detectable in low concentrations.

### Differential *TaCAD* expression after pathogen elicitation

**Fig. 6:**
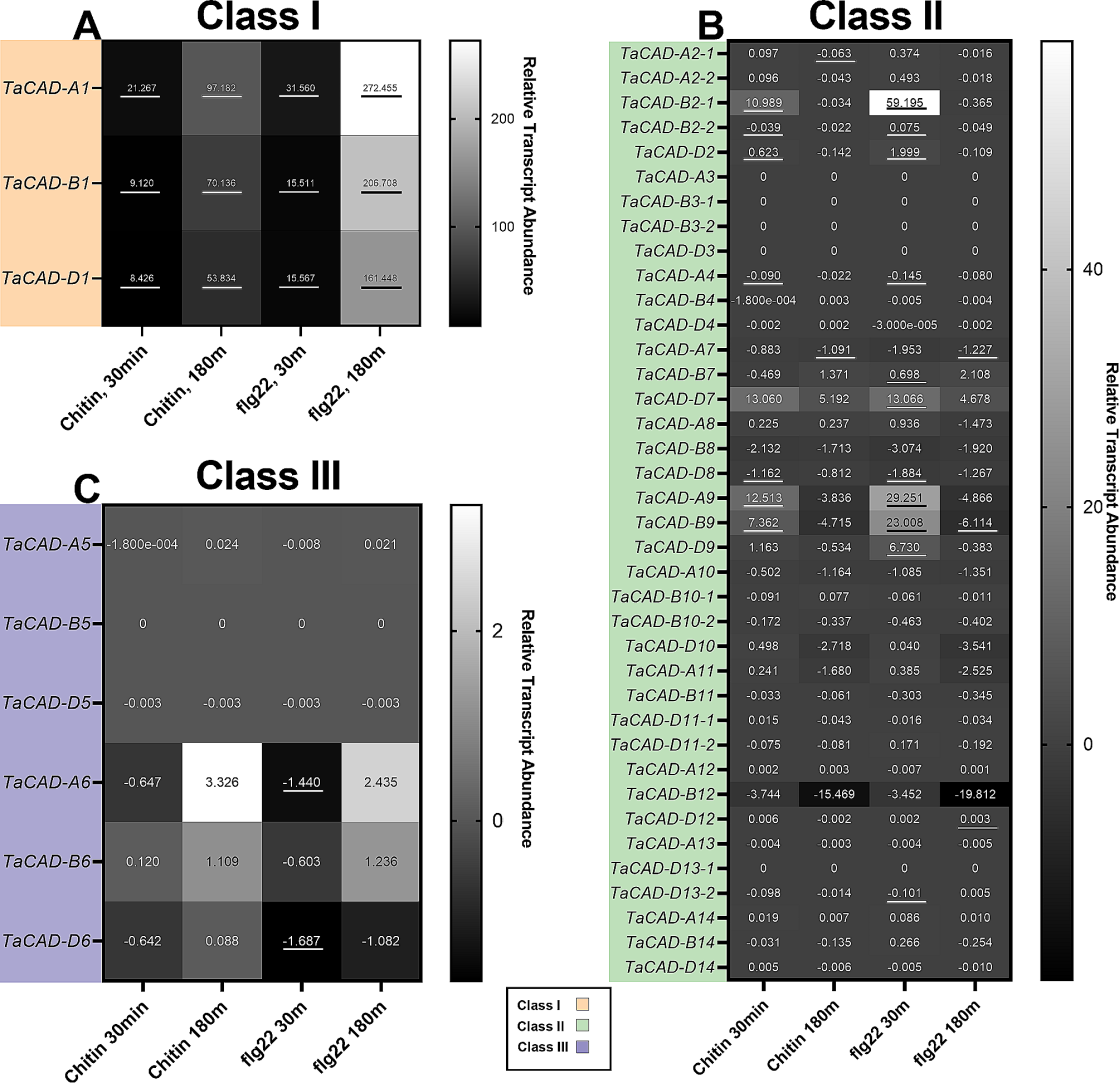
Heatmap of elicitor mediated differential TaCAD expression from Chinese Spring (CS) seedling leaf tissue. Values depicted are the differences between average values of three control samples versus three treatment samples. Significance was calculated using a TTest (*P* < 0.05) and is depicted by underlined values. (**A**) class I *TaCADs* appear to be significantly upregulated at both time points in response to both chitin and flg22. (**B**) *TaCAD-D2*, select *TaCAD-8* and select *TaCAD-9* homoeologs were observed to be significantly upregulated to chitin and flg22 at 30 min compared to control, but then are downregulated at 180 min. In general, class II *TaCAD* upregulation juxtaposes class I *TaCAD* differential expression. *TaCAD-12* homoeologs were not observed to be differentially upregulated (**C**) Differential Class III *TaCADs* appear to be expressed at low levels in response to chitin and flg22 in seedling leaf tissue

Past biotic stress studies have shown that pathogens trigger differential expression of phenylpropanoid genes. Therefore, the *TaCAD* gene family was analyzed for differential expression after pathogen elicitation in the CS genetic background. RNAseq data were analyzed from CS seedling leaf tissue after wounding and exposure to elicitors chitin (1 g/L), flg22 (500mM), or sterile water (mock) for either 30–180 min. A 4.5-fold increase in *TaCAD-A1*, 7.6-fold increase in *TaCAD-B1*, and a 6.3-fold increase in *TaCAD-D1* were observed after 180 min of chitin exposure to wounded leaf tissue (Fig. 6A). Interestingly, the bacterial elicitor flg22 caused an 8.6-fold increase in *TaCAD-A1*, 13.3-fold increase in *TaCAD-B1*, and a 10.3-fold increase in *TaCAD-D1* after leaf wounding and 180 min of flg22 exposure, compared to the 30-minute mark.

Most class II *TaCADs* were unresponsive to either chitin or flg22, with notable exceptions (Fig. 6B). *TaCAD-D2*, *TaCAD-D8*, and the *CAD-9* homoeolog triad were found to be significantly upregulated after 30 min of either chitin or flg22 treatment but were all shown to significantly decrease in transcript abundance after 180 min. Surprisingly, *TaCAD-A12* and its homoeologs showed no significant changes in expression after 30–180 min of chitin or flg22 treatment. Lastly, wounding and elicitor exposure produced no significant class III *TaCAD* transcriptional response compared to class I and some class II *TaCADs* in leaf tissue (Fig. 6C). Among the class III *TaCADs*, *TaCAD-A6* was found most expressed in response to both chitin and flg22 elicitation.

**Fig. 7:**
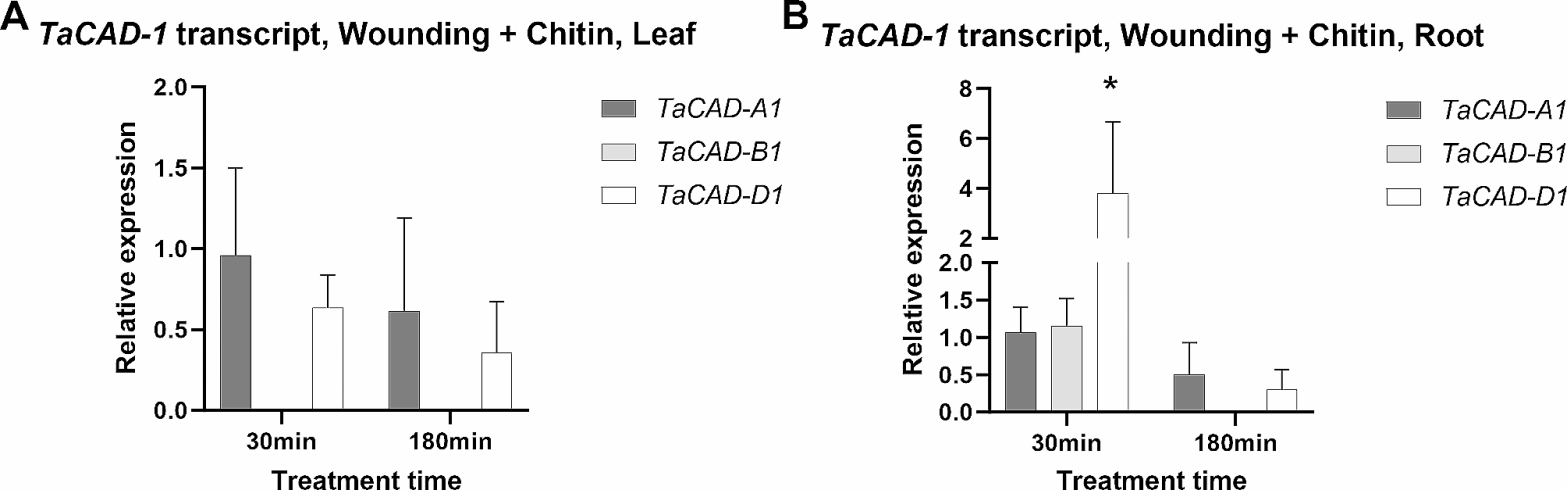
Class I *TaCAD* normalized differential expression after chitin exposure in leaf and root tissue. Transcript is relative to *GAPDH* and normalized to mock treatment (sterile water). (**A**) Insignificant downregulation of both *TaCAD-A1* and *TaCAD-B1* was observed at both 30 min and 180 min. (**B**) In contrast to unstressed tissue (Fig. 5B), all class I *TaCADs* were detectable in roots 30 min after treatment, and TaCAD-D1 was found to be significantly upregulated after 30 min of treatment

To validate the upregulation observed of the class I *TaCAD*s, the class I homoeolog-specific primers were used to quantify relative transcript abundance after leaf wounding and chitin treatment (Fig. 7A). In contrast the RNAseq data, class I *TaCAD* transcript in leaf tissue did not significantly change after wounding and chitin exposure (Fig. 7A). Although a significant increase in *TaCAD-D1* transcript in roots was detected 30 min after chitin exposure, both *TaCAD-B1* and *TaCAD-D1* transcript in roots appears to decrease 180 min after chitin elicitation (Fig. 7B).

### *Cis*-regulatory element abundance and CpG island distribution suggest multiple signaling pathways influence *TaCAD* expression

To evaluate what may drive *T*a*CAD* expression, putative *TaCAD cis*-regulatory elements were identified and characterized in both the 5’ UTR and 2000 bp upstream of the 5’ UTR. In general terms, all three classes *TaCAD* displayed very few *cis*-regulatory elements associated with cold, heat, or drought stress (Figure S2A). Conversely, the greatest number of *cis*-regulatory elements found among all three classes were related to light responsiveness, wounding, and pathogen responses. The predicted *cis*-regulatory elements within the class I *TaCAD* homoeolog promoter regions reflect generalized class I functions pertaining to growth and pathogen resistance. Light-responsive elements were found to be the most numerous predicted *cis*-regulatory elements in class I *TaCAD* promoter regions, followed by elements related to wounding and pathogens. Because of class II *TaCAD-A12* correlation with disease resistance, all three *TaCAD-12* homoeolog promoters were individually analyzed (Figure S4A). *TaCAD-A12* contains significantly more wounding and pathogen elements compared to its homoeologs and class I *TaCADs*. The homoeologs phylogenetically related to the *TaCAD-12* homoeologs, the *TaCAD-11* and *TaCAD-14*, also contain a high number of predicted wounding and pathogen-related *cis*-regulatory elements.

Phytohormone-related *cis*-regulatory elements were also analyzed to further determine how, when and where *TaCADs* may be expressed (Figure S4B). Notably, no *cis*-regulatory elements related to drought were detected in the *TaCAD-A1* promoter region, but among all class I *TaCAD* homeologs, the *TaCAD-A1* promoter region is predicted to contain the most ARBE *cis*-regulatory elements responsive to abscisic acid. Salicylic acid response elements were found to be the most numerous predicted phytohormone *cis*-regulatory elements in class I *CAD* promoter regions, whereas abscisic acid response elements were found to be the most numerous predicted phytohormone *cis*-regulatory elements in both class II and class III *TaCAD* promoter sequences. In contrast, both gibberellin and ethylene-related *cis*-regulatory elements were least present in all *TaCAD* promoter regions.

*Cis*-regulatory elements related to specific tissue types were also found throughout the analysis. For instance, *cis*-regulatory elements related to the development of the gynoecium, stamen, and leaf (CTAG-motif and HD-Zip 1) were found in class II *CADs TaCAD-D9* and *TaCAD-B12* as well as class III *TaCAD-B5* and *TaCAD-D5*. A *cis*-regulatory element related to root-specific oxidative stress (H-Box) was also predicted to be found in class II *TaCAD-D9*, and *TaCAD* promoters containing predicted *cis*-regulatory elements for xylem (AC-I) (*TaCAD-D9*), phloem (AC-II) (Ta*CAD-A12*), and meristem development (CAT-box, CCGTCC-box, re2f-1) (all class Is, many class IIs) were found. Finally, all *TaCAD* promoter regions except *TaCAD-D12* contained MYB binding-sites that may be associated with lignification (Figure S4C).

To determine the potential for epigenetic modulation of *TaCAD* transcription, CpG islands were predicted within *TaCAD* promoters and gene bodies (Table S5). CpG islands are predicted to be absent in all three class I *TaCAD* promoter regions, but multiple overlapping CpG islands were predicted within their gene bodies. Class II *TaCAD-8*, *TaCAD-9*, and *TaCAD-13* homoeologs contain predicted CpG islands in both promoter and gene body regions (Figure S4D). Ta*CAD-A12* was found to possess one more predicted CpG island in its defined promotor region compared to its putative homoeologs *TaCAD-B12* and *TaCAD-D12*. Class III *TaCAD-5* homoeolog triad contain several predicted CpG islands in their promoter regions, but no CpG islands were predicted in their gene bodies.

## Discussion

Analysis of the mechanisms that drive gene expression in hexaploid *T. aestivum* suggests that wheat homoeolog gene expression is complex and nuanced both between varieties and within variety populations. For instance, the polyploidization event of tetraploid (AABB) *Triticum turgidum* with the theorized D subgenome progenitor *T. urartu* was evolutionarily recent [74, 75], thus the probability of sub-functionalization is low [76]. Four types of homoeolog distribution (A: B:D, N:B: D, A: N:D, and A: B:N) account for 85% of all genes, with microsynteny among all subgenomes (A: B:D) accounting for 47% of homoeologs [77]. Moreover, variations in *cis*-elements and *trans*-acting effects amongst homoeologs significantly influence gene expression and downstream phenotypes [78, 79]. In addition, epigenetics plays a crucial role on gene regulation in wheat as only one homoeolog was found preferentially expressed due to either histone modification or transposable element modification in promoter regions amongst 30% of homoeolog triads [73]. Taken together, these data suggest multiple mechanisms likely regulate *CAD* expression.

We first postulated each of the subgenomes possess conserved *TaCAD* homoeologs. However, only *TaCAD1(TaCAD-D1)* [66, 67] and *TaCAD12* [62, 66, 67] have been identified and characterized. The rooted phylogenetic analysis presented in this study (Fig. 1) suggests 47 high confidence *TaCAD* gene copies are present in the reference genome of wheat (CS). The present study thus enhances the previous *TaCAD* investigations by providing the identities, sequences, and putative genomic locations of two additional homoeologs for *TaCAD-D1* and two additional homoeologs for *TaCAD-A12* that may be functionally significant. In addition, nine of the fourteen *TaCAD* homoeolog groups in the CS genetic background (64.2%) possess only one *TaCAD* copy from each subgenome (A: B:D), whereas four of the fourteen *TaCAD* homoeolog groups (28.5%) contain one or more phylogenetically determined duplicate, and one homoeolog group (7.2%) appears to be missing a *TaCAD* copy on the B subgenome (A: N:D) (Fig. 2A). The data presented in this study suggests the homoeolog distribution within the *TaCAD* family aligns with the recent homoeolog distribution analysis [77], thus supporting the validity of our phylogenetic organization of the *TaCAD* family.

Class I *CADs* are theorized to be the primary *CAD* copy expressed and catalytically active in lignifying tissues [64, 80, 81], and have been found co-expressed with other monolignol synthesis enzymes in *A. thaliana* [82], *P. virgatum* [83], *Eucalyptus grandis* [84], *Setaria viridis* [85], and *Populus tomentosa* [86]. As an additional quality control measure to ensure that the Class I *TaCAD* genes function in lignin biosynthesis we performed co-expression analysis with other lignin genes such as COMT, PAL, PTAL, and F5H (Figure S2).

Considering the significant role class I CADs contribute to plant growth, *TaCAD-A1*, *TaCAD-B1*, and *TaCAD-D1* received weighted attention in this study. Previous expression analysis of *TaCAD-D1* showed detectable transcript in stem and leaves, but not in roots four weeks after booting initiation (Ma 2010). To investigate if *TaCAD-D1* is preferentially expressed over *TaCAD-A1* and/or *TaCAD-B1* in the CS genetic background, transcriptomic profiles of the class I *TaCAD* triad were analyzed in different tissue types and development stages (Fig. 4). The low detectable TaCAD transcript (Fig. 4B) and poor co-expression with other phenylpropanoid synthesis-related putative functional orthologs in root tissues during all developmental stages (Figure S3) suggests *T. aestivum* roots are minimally lignified, and root lignification is likely limited to specific cell types such as the epidermis, endodermis, xylem, phloem, and interfascicular fibers [87]. Our qRT-PCR using CS homoeolog-specific class I *TaCAD* primers supports this postulation, as we were unable detect class I *TaCAD* transcript in root tissue during the vegetative and reproductive stages of development. Taken together, our findings support the previous identification and characterization of *TaCAD-D1* [66], and is congruent with past observations of wheat prioritizing aerial tissue growth over root growth after the vegetative phase of development [88].

The observed co-expression of *TaCAD-A1* and *TaCAD-D1* but absence of *TaCAD-B1* in later growth stages is likely due to epigenetic factors [73] and differences in *cis*-regulatory elements present the respective promoter regions [76, 79]. All three class I *TaCADs* possess predicted MYB, AC, and xylem/phloem-related transcription factor binding sites (Figure S4C), which may be directly controlled by and/or downstream from lignin transcription factors [89–92]. In contrast to all class II and class III *TaCADs*, all class I *TaCADs* homoeologs lack detectable CpG islands in their promotor regions, but all contain at least three conserved putative CpG islands throughout each homoeolog gene body (Table S4). While gene body methylation is likely not a dominant singular epigenetic control mechanism in *T. aestivum* [93] gene body methylation is correlated with active transcription in *T. aestivum* [94] and *O. sativa* [95].

*TaCADs* have been found to be co-expressed with other lignin synthesis genes in wheat lines resistant to *Fusarium graminearum* (Fusarium head blight) [96–99], *Rhizoctonia cerealis* (sharp eyespot disease) [62], and *Heterodera avenae* (cereal cyst nematode) [100]. Of particular note, several genes associated with lignin synthesis and deposition (*TaCAD*, *4-Coumarate*: *CoA ligase*, *callose synthase*, and *ABC transporter-4*) were identified within a QTL located on the short arm 6B chromosome of a *T. aestivum* line resistant to *F. graminearum* [98]. *TaCAD-B1* is the only *TaCAD* annotated on the short arm of the 6B chromosome (Fig. 2A), suggesting that TaCAD-B1 activity may partially contribute to *F. graminearum* resistance via a stress lignin deposition [21, 101]. In support of these findings, *cis*-regulatory elements related to salicylic acid signaling, jasmonic acid signaling, and wounding and pathogen responses are predicted to be in the *TaCAD-B1* promoter region (Figure S4B), and RNAseq data shows *TaCAD-B1* is upregulated after 30 and 180 min of chitin exposure in leaves (Fig. 6A). These data led us to postulate *TaCAD-B1* is upregulated in response to wounding and chitin exposure.

Although chitin triggered class I TaCAD expression via RNAseq [73], as we were unable to detect class I *TaCAD* expression using homoeolog-specific primers in our RT-qPCR experiments. In the RNAseq experiment, the leaves were incubated overnight and then subjected to the chitin elicitor [73]. We postulated that the overnight incubation affected our results by priming the stress response [102, 103]. Thus, tested if class I *TaCAD* expression is quickly upregulated without preliminary vacuum infiltration and overnight tissue incubation. The resulting data shows that only *TaCAD-D1* significantly increases in roots 30 min after chitin exposure (Fig. 7B), which may indicate that *TaCAD-D1* is part of a rapid stress signaling response [63]. These data suggest experimental and perhaps genotype as well as tissue-specific differences in the chitin elicitor response. In addition, time of pathogen exposure appears to be positively correlated with *TaCAD* expression in pathogen-resistant wheat lines [62, 98, 100]. Taken together, these data highlight that stress lignification may be a delayed stress responses and that there are genotypic differences in wheat lines [79, 104].

Expression data presented in this study suggest that class II TaCAD subunits may contribute a novel, uncharacterized function that modulates the rate of monolignol synthesis. Under unstressed conditions, both class II *TaCAD-8* and *TaCAD-9* homoeologs are expressed similarly to the class I *TaCADs* (Fig. 4), and class II *TaCAD-D9*,*TaCAD-A1* and *TaCAD-D1* are co-expressed in root tissue, but not in shoot tissue (Figure S4). Class I and II CAD co-expression has been previously observed in *S. bicolor* class II *SbCAD4* [36, 39] and *O. sativa* class II *OsCAD7* [32] and *OsCAD8A-D* [31, 54]. The class II TaCAD-8 and TaCAD-9 homoeologs are phylogenetically related to SbCAD4 and OsCAD8A-D (Fig. 1) and possess remarkably similar primary sequences (Fig. 3B). SbCAD4 has been shown to be catalytically active towards cinnamaldehydes but displays significantly slower substrate turnover rate quantified versus class I SbCAD2 [39, 105], leaving its metabolic purpose unclear.

However, inverse expression profiles of the *TaCAD-8* and *TaCAD-9* homoeologs (Fig. 6B) relative to the class I *TaCAD* homoeologs (Fig. 6A) suggest these class II may modulate an uncharacterized biotic stress signaling function. Recent reports of class I and class II CADs physically interacting with and influencing the activity of other genes and enzymes suggest class I and class II TaCADs may also be sub-functionalized beyond their known catalysis of cinnamaldehyde dehydration. For instance, *Populus trichocarpa* class I PtrCAD1 was observed to both physically interact with cinnamoyl CoA reductase (CCR) PtrCCR1, forming CAD/CCR heterodimers that modulated CCR enzymatic activity [106]. Furthermore, perturbation of either *PtrCAD1* or *PtrCCR1* resulted in downregulation of the other gene, suggesting a transcriptional feedback loop regulates lignin gene expression. Similarly, significant overexpression of *TaCAD-A12* led to a significant increase in *TaCAD1*,* TaCCR*, *TaCOMT1*,* Defensin*,* PR10*,* PR17c*, and *Chitinase1* expression [62]. Lastly, *A. thaliana* class II *AtCAD7* was recently characterized as an S-gene after pathogen effectors were observed to physically interact with AtCAD7, resulting in increased pathogen susceptibility [63]. Taken together, these data provide evidence that CAD subunits do not exclusively form homodimers, and that class I and class II CAD subunits attune both transcription and enzyme activity outside of their NADPH-mediated cinnamaldehyde dehydration catalysis. Future analysis of class I and class II TaCAD protein-protein interactions may reveal uncharacterized signaling pathways that regulate responses to biological and environmental stressors.

## Conclusion

As accessibility, quality, and scope of large-scale datasets increase, so too does the discovery of complex regulatory relationships that govern favorable crop phenotypes. This study is a product of high-quality public datasets, expanding on previous studies while simultaneously laying foundation for future research. The identification and characterization of the *TaCAD* gene family presented in this study provide much-needed basic data that future functional studies can leverage to optimize their selection for lignin stress-responsive phenotypes. Moreover, this study highlights the complexity and redundancy present in hexaploid bread wheat that likely aids in both its prolific habitat range and its robust resilience to a broad range of biotic and abiotic stresses. Future research in the area of lignin biosynthesis will contribute to improving wheat breeding strategies that lead to bolstering yields in the face of climate change.

## Materials and methods

### Identification of the *CAD* gene family in wheat

The class I CAD *Brachypodium distachyon* protein sequence (Bradi3g06480) named as both BdCAD5 [28] and BdCAD1 [28–30] was used in a BLAST to query the *T. aestivum sp.* Chinese spring (RefSeqv1.0) proteome on the Ensembl Plants database [107, 108]. The BLAST parameters were an E-value greater than 1e^− 1^ and identity similarity > 30%. Utilizing the *BdCAD1* genomic sequence, an additional BLASTn search was also performed in the *T. aestivum* genome to search for low confidence *T. aestivum* paralogues. A final recursive pBLAST search for any remaining TaCAD isoforms was performed utilizing all 47 TaCAD proteomic sequences to rule out potential sequence bias. Lastly, the *BdCAD1* genomic sequence was queried for *CAD* and *CAD-like* orthologs in *Triticum urartu* (A subgenome progenitor), and *Aegilops speltoides* (B genome progenitor model scaffold), and *Aegilops tauschii* (D subgenome progenitor) [72].

### Phylogenetic analysis and *TaCAD* naming

Two hundred and nineteen amino acid sequences of CAD proteins from the species/sub-genomes listed (Table S3) were aligned using MUSCLE 3.7 [109] as implemented through the CIPRES Science Gateway [110]. A diverse set of species derived from gymnosperms, eudicots, and monocots was chosen to root the phylogeny. The amino acid alignment was analyzed using maximum likelihood (ML) with RAxML-HPC BlackBox (8.2.12) [111] in CIPRES implementing the PROTCATJTT + G model with 1000 rapid bootstrap inferences and a thorough ML search. Trees were rooted between the class II CAD clade and the class I + class III CAD clade.

Utilizing phylogenetic homoeolog associations, *TaCADs* are named under the current *T. aestivum* naming guidelines [112]. All *TaCADs* homoeologs are differentiated by subgenome (ex: *TaCAD-A2-1* versus *TaCAD-B2-1*); numbered in the order by which they appear in the genome (ex: *TaCAD-A2-1* versus *TaCAD-A3*); and duplicate genes, determined by both phylogenetic relationship and approximate physical relative proximity, are denoted by a hyphenated numerical designator at the end of the gene name (ex: *TaCAD-A2-1* versus *TaCAD-A2-2*). Two exceptions were made to this naming convention: *TaCAD1* (GU563724, TraesCS6D02G162800) was designated *TaCAD-D1* to preserve its original designation while also including its subgenome annotation (D subgenome) to differentiate *TaCAD-D1* from its homoeologs *TaCAD-A1* (TraesCS6A02G173700) annotated on the A subgenome, and *TaCAD-B1* (TraesCS6B02G201600) annotated on the B subgenome. Similarly, *TaCAD12* (TraesCS7A02G350500) was designated *TaCAD-A12* to distinguish it from its homoeologs *TaCAD-B12* (TraesCS7B02G232400) and *TaCAD-D12* (TraesCS7D02G328300).

### Gene and protein structure analysis

All *TaCAD* gene data pertaining to total gene length, transcript length, coding sequence length, number of exons, number of introns, and predicted number splice variants were collected from Ensembl Plants database [108] and summarized in table format. The annotated chromosomal locations of all *TaCADs* were then approximated on a chromosome map to visualize gene location relative to other approximated *TaCAD* annotations. Within this gene map, homoeologous relationships highlight likely chromosomal rearrangements. The intron and exon regions of all *TaCAD* genes were assembled utilizing the Gene Structure Display Server [113, 114]. Resulting gene structures were grouped together by phylogenetic classification, and subsequently organized by homoeologous association.

In addition, all TaCAD residue lengths, molecular weights, theoretical isoelectric points, theoretical charges, obtained from Ensembl Plants database [108] were summarized in table format. Predictive modeling was applied to all TaCAD sequences to estimate subcellular localization probability using the Bologna Unified Subcellular Component Annotator [115, 116]. Protein sequences of all class I TaCADs were aligned phylogenetically associated class I CADs using Clustal Omega ClustalW sequence alignment tool [117, 118]. This process was repeated with class II CAD subclade containing SbCAD4 [39] and TaCAD-8 and TaCAD-9 homoeolog groups, the subclade containing TaCAD-12 homoeolog group, and the class III subclade containing both TaCAD-5 and TaCAD-6 homoeolog triads.

### Promoter and CpG island analysis

In this study, the promoter of any gene includes both the 5’ UTR as defined by the Ensembl Plants *T. aestivum* database and 2000 base pairs upstream of the 5’ UTR [108]. The promoter regions were queried in the PlantCARE promoter element database [119]. In addition, promoter and gene body methylation potential was determined via CpG island prediction using EMBOSS CpGplot [120, 121].

### RNAseq analysis

All transcriptomic data were collected from a publicly accessible high fidelity transcriptomic data from the expVIP Wheat Expression Browser [73, 122]. *TaCAD* TPM values in specific *T. aestivum* sp. Chinese Spring organs at seedling, vegetative, and reproductive stages of development in roots, leaves, nodes, and spikelets were used to generate heat maps using GraphPad Prism 10.2 to visualize total *TaCAD* transcript abundance. Phenylpropanoid synthesis-related putative functional ortholog genes were identified via closest protein sequence identity match to functionally characterized *B. distachyon* orthologs [27]. TPM values of these putative functional orthologs were then collected from the expVIP Wheat Expression Browser and subjected to a hierarchical clustering analysis via iDEP [123]. Lastly, the expression ratios of the *TaCAD* family in 21dag wounded leaves after chitin or flg22 elicitation were used to generate additional heatmaps to visually compare stress-induced transcription patterns within *TaCAD* classifications [124, 125]. Significance was calculated using an unpaired double tailed TTest with (*P* < 0.05).

### Plant growth conditions

*Triticum aestivum* L. subsp. *aestivum* cv. Chinese Spring was obtained from GRIN-Global Project. Seedlings were grown in a well-watered 2:1 Sunshine #4 (Sungro Horticulture, Agawam, MA, USA) and Turface (Turface Athletics, Buffalo Grove, IL, USA) substrate mixture supplemented with 14:14:14 Osmocote™ fertilizer (ScottsMiracle-Gro, Marysville, OH, USA) at a consistent 22˚C temperature with a 16:8 light schedule. Tissue samples were harvested and immediately flash frozen in liquid N_2_ 10 h after first light at timepoints closely reflecting RNAseq timepoints, defined as Feekes growth stages (1, 3, 6, 8, and 10.5). For the chitin response assay, the PAMP-triggered immune response assay used to generate the transcriptomic dataset was partially replicated as previously described [73] with the following modifications: whole 2nd and 3rd leaf were wounded via scraping with a spatula, and then were immediate submerged in either mock (sterilized ddH_2_O) or treatment (1 g/L chitin (Sigma-Aldrich, St. Louis, MO, USA) boiled and suspended in room temperature sterilized ddH_2_O) for 30–180 min with no rest period or vacuum infiltration. For root chitin treatment, plants were removed from growth media, wounded with a spatula, and transferred into to sterile blacked out 50mL conical tubes containing mock (sterilized ddH_2_O) or treatment (1 g/L chitin) for 30 min and 180 min with no rest period or vacuum infiltration. After treatment, both leaf and root tissue were immediately flash frozen in liquid N_2_ and stored at -80˚C until further processing.

### RNA purification

All tissues were homogenized with clean mortar and pestle under N_2_. RNA was extracted using TRIzol™ reagent (Thermo Fisher Scientific, Waltham, MA, USA) following manufacturer’s recommended protocol with no modifications and quantified on an Eppendorf Biospectrometer^®^. Purified RNA was additionally processed with an Invitrogen Turbo DNAseI kit (Thermo Fisher Scientific, Waltham, MA, USA) to remove trace contaminating genomic DNA.

### Quantitative real Time-PCR

To probe for class I *TaCAD* homoeolog-specific transcripts, primers were designed based on the *Triticum aestivum sp* Chinese Spring (CS) reference genome within unique insertion-deletion regions in the 3’ UTRs of the three class I *TaCADs* (Table S5). cDNA was generated utilizing the iScript™ Reverse Transcription Supermix for RT-qPCR (Bio-Rad, Hercules, CA, USA) and protocol modified with the addition of 0.5 M of betaine (Sigma-Aldrich, St. Louis, MO, USA) added per reaction. To perform qRT-PCR, SsoAdvanced™ Universal SYBR^®^ Green Supermix (Bio-Rad, Hercules, CA, USA) was used following manufacturer’s protocol, and Cq values were obtained using a Bio-Rad CFX96 Real-Time System reader. Primer specificity was successfully validated on CS cDNA using a melt curve and running gel electrophoresis with a 2% agarose gel on the PCR products. Subsequent qRT-PCR was performed on three biological replicates, with three technical replicates per biological replicate. The housekeeping gene used was *glyceraldehyde-3-phosphate dehydrogenase* (*GAPDH*) (Table S5). Relative expression was calculated utilizing the 2^(−ΔΔCt)^ as previously described [126], and normalized to the untreated control. Significance of the transcript differentials observed were calculated using an unpaired double-tailed TTest (*P* < 0.05).

### Electronic supplementary material

Below is the link to the electronic supplementary material.

Supplementary Material 1

Supplementary Material 2

Supplementary Material 3

Supplementary Material 4

Supplementary Material 5

Supplementary Material 6

## Data Availability

All data generated or analyzed during this study are included in this article and its supplementary material. The TaCAD protein sequences named in this study have been deposited in GenBank and the GenBank identification numbers can be found in Supplementary Table 2.

## Declarations

All plant material used in this study was obtained from GRIN-global (grin-global.org).

## Abbreviations

ABC transporter-4: ATP-binding cassette transporter-4
BUSCA: Bologna Unified Subcellular Component Annotator
CAD: Cinnamyl alcohol dehydrogenase
CCR: Cinnamoyl CoA reductase
cDNA: Coding DNA
COMT: Caffeic acid 3-O-methyltransferase
CS: Chinese Spring
DAG: Days after germination
ddH2O: Double distilled water
Flg22: Flagellin 22
G: Coniferyl alcohol
GAPDH: Glyceraldehyde-3-phosphate dehydrogenase
H: p-Coumaryl alcohol
ML: Maximum likelihood
NAD(H): Nicotinamide adenine dinucleotide
NADP(H): Nicotinamide adenine dinucleotide phosphate
PAMP: Pathogen associated molecular pattern
PR10: Pathogenesis-Related 10
PR17c: Pathogenesis-Related 17c
qRT-PCR: Quantitative real-time polymerase chain reaction
QTL: Quantitative trait loci
RNAseq: RNA sequencing
S: Sinapyl alcohol
S-gene: Susceptibility gene
UTR: Untranslated region
Zn^2+^: Zinc
