# Supplementary figures and images for: Genome-wide characterization and expression analysis of the CINNAMYL ALCOHOL DEHYDROGENASE gene family in Triticum aestivum
Source: BMC Genomics. 2024 Aug 29;25:816. doi: 10.1186/s12864-024-10648-w (PMC11363449; doi:10.1186/s12864-024-10648-w)

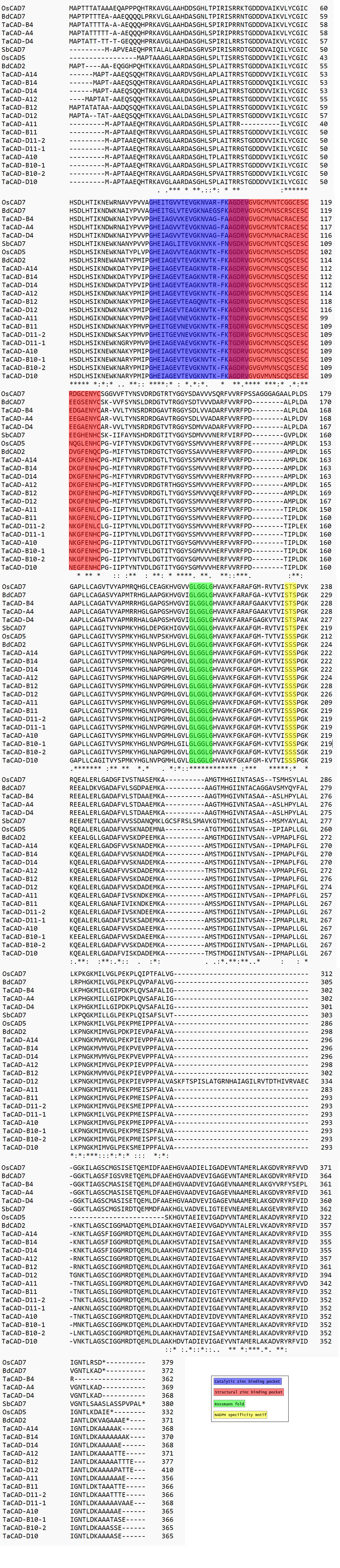

Supplement: Supplementary file 1 — Supplementary Material 1 [file 12864_2024_10648_MOESM1_ESM.tif]

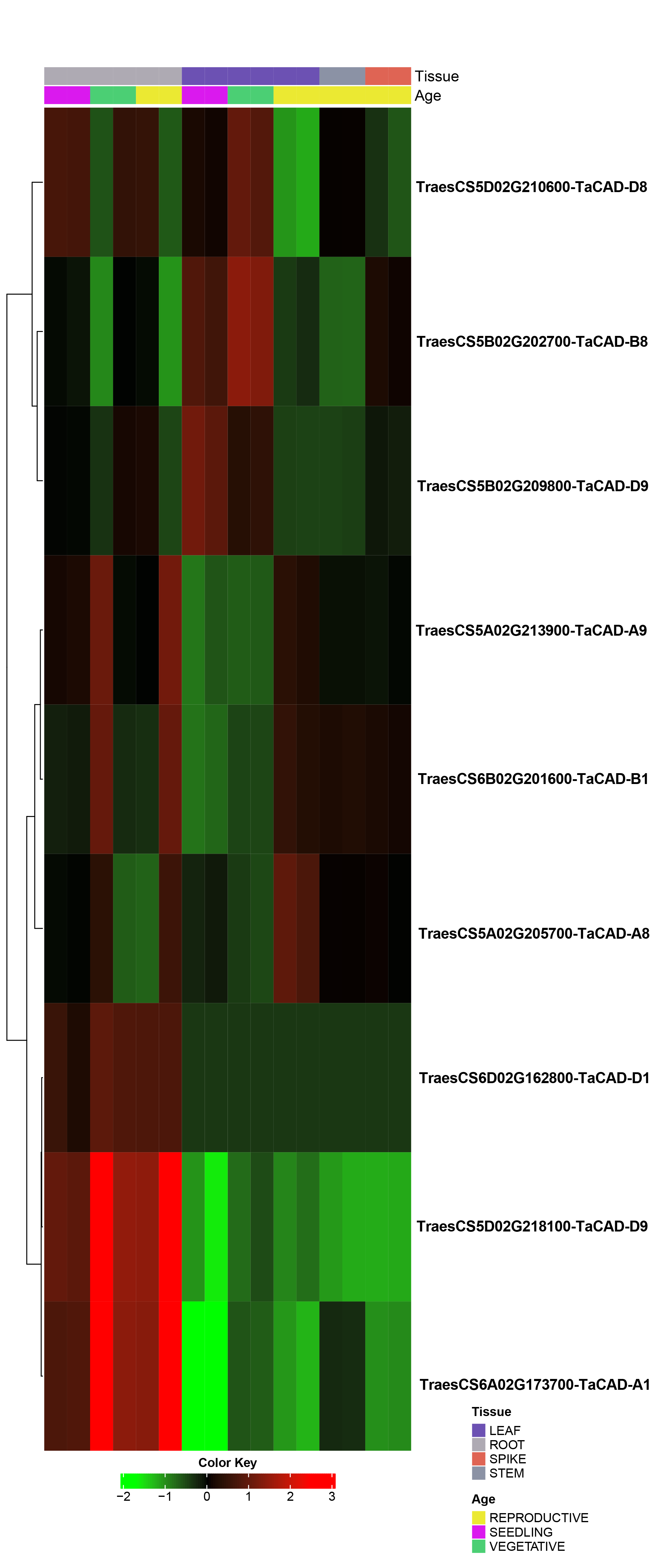

Supplement: Supplementary file 2 — Supplementary Material 2 [file 12864_2024_10648_MOESM2_ESM.tif]

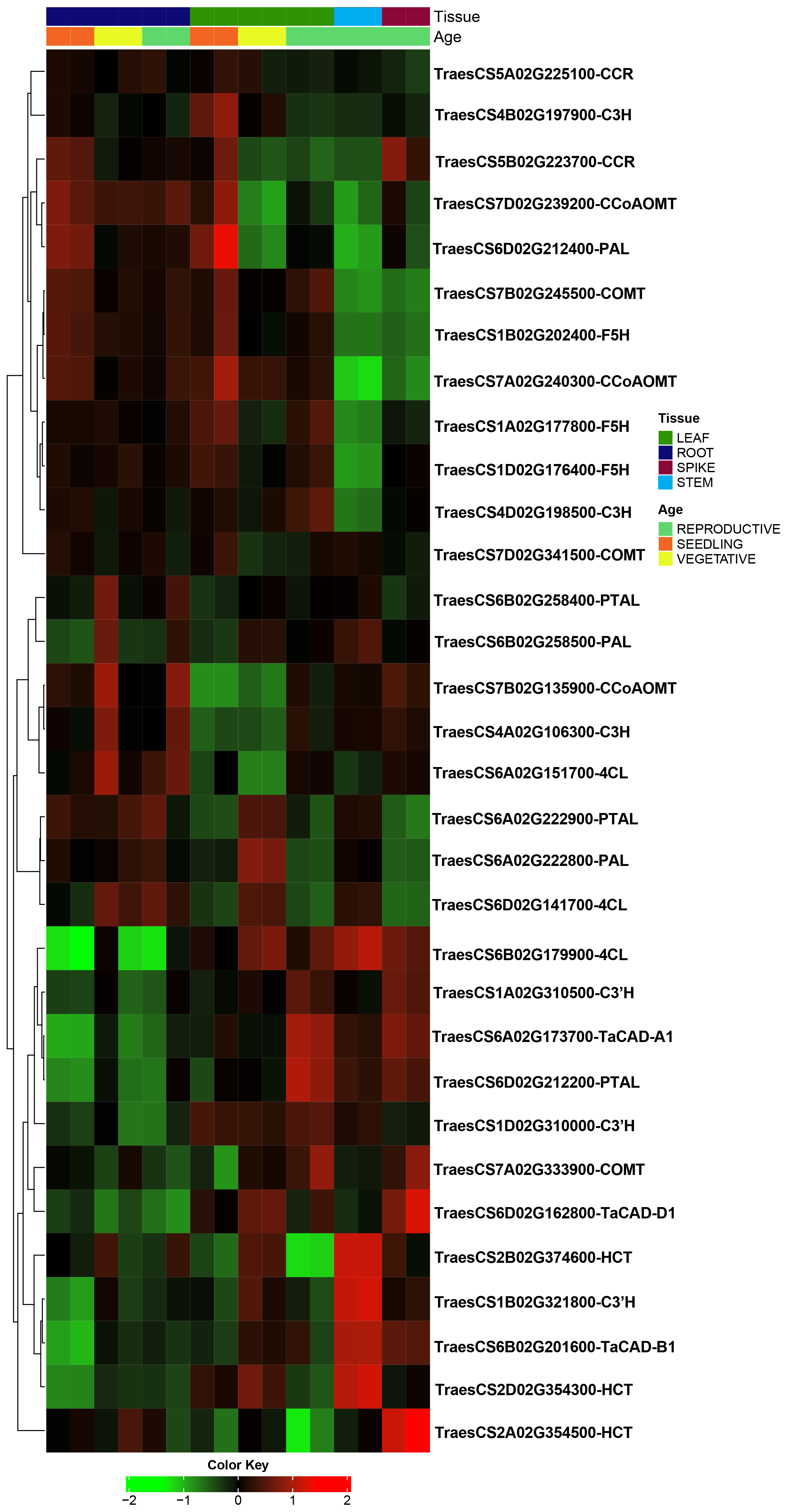

Supplement: Supplementary file 3 — Supplementary Material 3 [file 12864_2024_10648_MOESM3_ESM.tif]

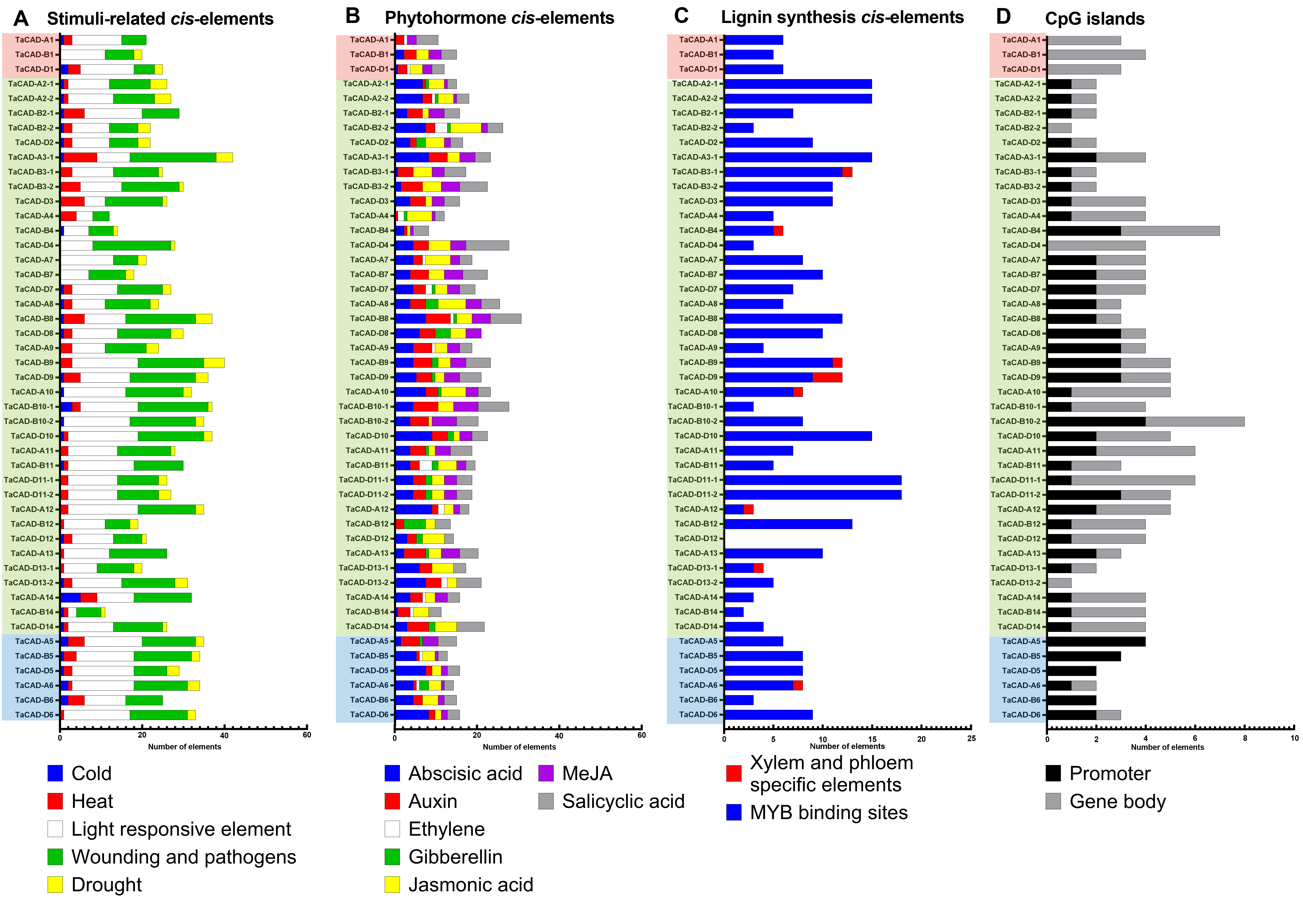

Supplement: Supplementary file 4 — Supplementary Material 4 [file 12864_2024_10648_MOESM4_ESM.tif]
